# Parental acceptance of Silver Diamine Fluoride in two lower-middle-income countries: Iran and Tajikistan

**DOI:** 10.1186/s12903-024-04434-z

**Published:** 2024-06-13

**Authors:** Sedigheh Sabbagh, Sara Moradi, Gelareh Haghi-Ashtiani, Gulomnabi Bakhtibekov, Samira Manaseki-Holland, Vahid Ravaghi

**Affiliations:** 1 Dental Research Center, Research Institute of Dental Sciences, Shahid Beheshti University of Medical Sciences, Tehran, Iran; 2 Department of Health Research Methods, Evidence, and Impact, McMaster University, Hamilton, ON Canada; 3 Institute of Dentistry, Barts and The London School of Medicine and Dentistry, Queen Mary University of London, London, UK; 4 Aga Khan Health Service, Dushanbe, Tajikistan; 5 Institute of Applied Health Research, College of Medical and Dental Sciences, University of Birmingham, Birmingham, B15 2TT UK; 6 School of Dentistry, University of Birmingham, Birmingham, UK

**Keywords:** Parental acceptance, Silver diamine fluoride, Dental caries, Child

## Abstract

**Background:**

Using Silver Diamine Fluoride (SDF) may be an effective public health approach for managing dental caries in children. Parental acceptance of SDF has rarely been investigated in low-income and middle-income countries (LMICs). The aim of this study was to evaluate parental acceptance of SDF to manage dental caries in children aged 2–12 in Iran and Tajikistan.

**Methods:**

This cross-sectional study was conducted in the Kurdistan province of Iran and Khatlon region of Tajikistan, 2022–2023. Parents watched a video about SDF and its weaknesses and strengths as compared to conventional approaches before completing the questionnaire. We also reported Prevalence Ratios with 95% confidence intervals for the relationship between parental acceptance and associated demographic factors as well as dental attitude and experience.

**Results:**

Participants were 245 and 160 parents in Iran and Tajikistan, respectively. In both countries, a majority (Iran: 61.6%, Tajikistan: 77.9%) accepted SDF over conventional treatments for all primary teeth. The majority also accepted SDF only for posterior permanent teeth (Iran: 73.5%, Tajikistan: 78.7%). Black discoloration was the main reason for rejecting SDF. Overall, demographic factors and dental experience and attitude were not significantly associated with SDF acceptance.

**Conclusions:**

SDF was widely accepted by Iranian and Tajik parents. Establishing parental acceptance of SDF is an important step toward its application in LMICs where inexpensive solutions are needed.

## Background

Dental caries is the most common human disease affecting billions of people in the world [1, 2]. Despite the overall decline in the prevalence of dental caries in high-income countries, dental caries appears to be on the rise in many low-income and middle-income countries (LMICs) [1]. Dental conditions such as caries, if they remain untreated, cause pain and discomfort and negatively affect quality of life [3]. A recent review study also has linked childhood dental caries to undernutrition [4]. Further, untreated dental caries costs societies both in terms of direct costs of treatments as well as productivity loss for parents of children with caries [5].

Conventional methods of treating dental caries typically involve anesthetizing the affected teeth, drilling the carious tissues, and replacing the cavity with filling materials/indirect restorations. Such treatments are often carried out by dentists using expensive equipment. Several limitations are associated with the conventional ‘drill and fill’ approach. From the clinical perspectives, the process of delivering such treatments may involve experiencing pain and discomfort [6]. Further, the equipment which is often required for conventional treatments is not available in low-resource geographies and communities. There are, however, alternative minimally invasive approaches which may not require removing tissue and dental injection [7].

Application of Silver Diamine Fluoride (SDF) is an example of an emerging minimally invasive approach. The SDF is a clear ammonia solution containing silver and fluoride and may be used for arresting active dental caries [8]. While SDF was first adopted in the 1960s in Japan [8] it has only recently realized its potential after it was cleared by the United States Food and Drug Administration [9, 10]. In recent years, there has been a wealth of evidence emerging from randomized controlled trials and multiple systematic reviews to support the effectiveness of SDF in arresting dental caries. A recent umbrella review, for example, summarised the findings of 11 systematic reviews and established the effectiveness of SDF in arresting caries affected primary teeth [11]. Such strong evidence in favour of SDF has encouraged global health policy makers to consider its potential for managing the widespread problem of dental caries. For example, SDF was endorsed for managing childhood dental caries by the 2017 World Health Organization (WHO) consultation report [12] as well as in the 2019 WHO implementation manual for tackling childhood dental caries [13]. In 2021, the report of the WHO expert committee listed SDF as an essential medicine. This document suggested the use of SDF in “non-specialized settings in alignment with WHO guidance on oral health interventions” [14]. The application of SDF, however, has yet to be adopted widely in dental education as well as national regulations [15, 16]. The fact that the SDF leaves black stains on teeth and its impact on parental perception seems to deter the wider application of SDF [17]. Establishing parental acceptance of SDF, therefore, is a fundamental step toward its wider promotion and application among children who have the highest burden of caries [18]. Several studies have been published, mostly in high-income and upper-middle-income countries, to report the parental acceptance of using SDF [19] while data is scarce from lower income countries [17].

The primary aim of this study was to examine parental acceptance of using SDF for managing childhood dental caries in two LMICs, as classified by the World Bank [20]: Iran and Tajikistan. The collaborative research team consists of researchers from both countries. Iran and Tajikistan not only are similar in terms of being LMICs, but also share similar culture. In addition to parental acceptance of using SDF, we also investigated the reasons for accepting or declining SDF treatment, and the demographic factors and dental attitude/experience which may predict parental acceptance.

## Methods

This was a cross-sectional study of parents which collected data in two countries. The present study was guided by STROBE guidelines [21]. Ethical approval was obtained from relevant authorities in both Tajikistan and Iran. Parents who participated in this study were provided with an information sheet and consented to participation prior to data collection.

### Sample size

The objective of this cross-sectional study was to report the acceptability of using SDF. Taking the previously reported rates of parental acceptance of SDF [18, 22], we determined that a sample of at least 150 study participants was necessary in each country with a two-tailed, type 1 error rate of 5% and a power of 80%. To account for a 15% non-response, we targeted a slightly higher number of participants at each data-collecting site.

### Participants and setting

This research was carried out in two LMICs in the Middle East and Central Asia: Iran and Tajikistan. Samples were recruited between June 2022 and January 2023. In Iran, we collected data from Kurdistan which is a western province of the country where most of the population speaks Kurdish. In Tajikistan, we collected data from Khatlon region which is one of the four provinces of Tajikistan where Tajik is the main language. In both Iran and Tajikistan, eligible participants were parents who had at least one child aged 2 to 12 years. Parents who had more than one eligible child were asked to report their acceptance of using SDF for their youngest child. If both parents were present, only one was asked to take part in the survey. Participants were recruited through convenience sampling. There were some inter-country differences in the settings where we collected data. In Iran, we collected data from parents who, for any reason, visited two government funded primary health care centres in two cities of the province of Kurdistan (Sanandaj and Saqqez). In Tajikistan, parents were recruited from five schools in five sites across the Khatlon region (Kulob city, Baljuvon, Khovaling, Temurmalik and Shamsiddini Shoin). In Iran, a trained primary health care worker showed the video and collected data through interviewing in an examination room at each primary health care centre. In Tajikistan, self-administered questionnaires were used, and the parents watched the video on a large screen. We did not collect clinical data from either parents or children in this study.

### Variables

The outcome measures of this study were the parental acceptance of using SDF for (1) primary, and (2) permanent dentitions. We asked parents whether they would be happy with using SDF for treating their youngest child’s primary/permanent teeth, irrespective of the treatment cost. Parents could report their agreement with using SDF for (a) all cavitated teeth, (b) only for front (visible) cavitated teeth, (c) only for back (invisible) cavitated teeth, and (d) none of the cavitated teeth. Parents who agreed with using SDF, at least once, were asked to indicate the strengths of SDF which made them decide to accept SDF. On the other hand, parents who did not agree with SDF for either primary or permanent dentition, were asked to indicate the weaknesses of SDF which prompted them to decline SDF. In addition to these, we also obtained data on demographic factors as well as dental experience and attitude to examine whether these predict the parental acceptance of SDF.

### Questionnaire and video

We developed and used two instruments in this study: (1) a four-minute video to give information on SDF to parents and (2) a questionnaire. Parents were invited to watch the video (in local language) before completing the questionnaire. In this video, the following topics were covered: (a) development of primary and permanent dentitions, (b) clinical appearance and consequences of (untreated) dental caries in children, (c) conventional methods of treating dental caries in children (i.e. performing tooth fillings and using prefabricated crowns), (d) SDF treatment in children. For both conventional and SDF treatments, we provided an overview of the materials/restorations applied, clinical procedures, and strengths and weaknesses using relevant videos and pictures. We also showed the before-and-after pictures of treated anterior and posterior teeth for each treatment in our video.

We followed a thorough process for preparing the study instruments. The video was produced using evidence-based sources such as guidelines from the American Academy of Pediatric Dentistry on SDF [23]. The questionnaire was designed to align with the information presented in the video. We also formed an independent expert panel consisting of five academic specialist pediatric dentists who had clinical experience of using SDF for children. This panel was consulted to ensure the accuracy as well as the content and face validity of the information in the video, including the strengths and weaknesses of SDF (Table 1), and the questionnaire. The wording and appropriateness of the questionnaire was checked with native speakers with previous experience of health and dental health research in their local areas. Moreover, both questionnaire and the content of the video were validated and piloted in a group of five parents in each country. The intra-rater reliability, in which the same rater completed the questionnaire in two weeks, was established through 100% agreement between the first and second response for all respondents.

### Statistical methods

Data analyses were carried out using STATA 18. We presented a frequency table to describe the sample and reported the rates of parental acceptance and Prevalence Ratios (PRs) with 95% confidence intervals (CI) for the relationship between the outcome variable (i.e. parental acceptance) and potential predictors.

**Table 1:** Strengths and weaknesses of SDF

Weaknesses	Strengths
(1) black discoloration	(1) no need for injection
(2) chemical composition	(2) no pain and no anxiety
(3) teeth remaining unrestored	(3) no drilling
(4) the possibility of repeating the treatment	(4) fast and easy

## Results

This study reported data from 245 to 160 parents in Iran and Tajikistan, respectively (Table 2). Participants in two countries varied in terms of their demographic factors. For example, four out of ten participants in Iran (40.8%) were male whilst all participants in Tajikistan were female. Further, the majority of participants in Tajikistan (64.4%) resided in rural areas, unlike Iran (24.5%). Participants in the two countries also differed in terms of dental experience and attitude. The majority of children in both Iran (71.4%) and Tajikistan (62.9%) had never visited a dentist. In terms of attitudes, seven out of ten Tajik participants felt that decayed primary teeth are not worth treating compared to nearly one third of participants (30.2%) in Iran.

**Table 2:** Sample characteristics

	Iran		Tajikistan	
	Frequency	%	Frequency	%
**Demographic characteristics**				
** Parent’s sex**				
Female	145	59.2	160	100.0
Male	100	40.8	0	0.0
** Place of residence**				
Urban	185	75.5	57	35.6
Rural	60	24.5	103	64.4
** The youngest child’s sex**				
Female	112	45.7	79	49.4
Male	133	54.3	81	50.6
**Dental experience and attitude**				
** Children’s experience of abscess/infection**				
Yes	159	65.7	63	45.3
No	85	34.8	76	54.7
** The youngest child’s experience of dental treatment**				
Yes	70	28.5	53	37.1
No	175	71.4	90	62.9
** Children’s primary teeth**				
Are not worth treating/ should be removed	74	30.2	94	69.6
Should be filled	171	69.8	41	30.3

Parental acceptance of using SDF is reported in Table 3. The majority of parents in Iran (61.6%) and Tajikistan (77.9%) were in favour of using SDF for all primary teeth. Also, the vast majority of parents supported the application of SDF only for posterior permanent teeth (73.5% in Iran and 78.7% in Tajikistan). Nearly all participants (between 90.8% and 100%) who agreed with the use of SDF for either primary or permanent dentition indicated all four strengths of SDF affected their decision. Among those who did not approve SDF for either primary or permanent teeth, black stain was the main reason for declining SDF.

**Table 3:** Parental acceptance of using silver diamine fluoride (SDF)

	Iran		Tajikistan	
	Frequency	%	Frequency	%
**Parental acceptance of SDF treatment**				
** Agreement with SDF for carious primary teeth**				
For all teeth	151	61.6	106	77.9
Only for front (visible) teeth	2	0.8	18	13.2
Only for back (invisible) teeth	89	36.3	5	3.7
For none	3	1.2	7	5.2
** Agreement with SDF for carious permanent teeth**				
For all teeth	12	4.9	6	4.4
Only for front (visible) teeth	1	0.4	16	11.8
Only for back (invisible) teeth	180	73.5	107	78.7
For none	52	21.2	7	5.1
**I chose SDF because…**				
** There is no injection**				
Yes	241	100.0	117	92.1
No	0	0.0	10	7.9
** There is no pain/no anxiety**				
Yes	241	100.0	108	90.8
No	0	0.0	11	9.2
** There is no drilling**				
Yes	241	100.0	98	95.2
No	0	0.0	5	4.8
** It is fast and easy**				
Yes	241	100.0	123	99.2
No	0	0.0	1	0.8
**I did not choose SDF because …**				
** Black stain**				
Yes	48	96.0	39	84.8
No	2	4.0	7	15.2
** Chemical composition of SDF**				
Yes	4	8.0	31	75.6
No	46	92.0	10	24.4
** Not properly filled (teeth remaining unrestored)**				
Yes	35	70.0	34	77.3
No	15	30.0	10	22.7
** Possibility of need for repeating treatment**				
Yes	11	22.0	29	82.8
No	39	78.0	6	11.2

Table 4 reports the PRs for the relationship between parental acceptance of using SDF and potential demographic factors, dental attitudes and experiences. None of the investigated factors were statistically related to parental acceptance.

**Table 4:** Factors associated with the parental acceptance of using silver diamine fluoride for all primary teeth and only posterior permanent teeth of children in Iran and Tajikistan

	Iran				Tajikistan			
	All primary teeth		Posterior permanent teeth		All primary teeth		Posterior permanent teeth	
	Rate	PR (CI)	Rate	PR (CI)	Rate	PR (CI)	Rate	PR (CI)
**Demographic factors**								
** Parent’s sex**								
Female	61.4	1	73.8	1	77.9		78.7	
Male	62.0	1.01 (0.73, 1.40)	73.0	0.99 (0.73, 1.33)	0.0	N/A	0.0	N/A
** Place of residence**								
Urban	56.2	1	70.3	1	72.3	1	83.7	1
Rural	78.3	1.39 (0.99, 1.97)	83.3	1.19 (0.86, 1.64)	80.9	1.12 (0.74, 1.68)	75.9	0.91 (0.61, 1.34)
** The youngest child’s sex**								
Male	60.9	1	72.9	1	82.1	1	77.9	1
Female	62.5	1.03 (0.75, 1.41)	74.1	1.02 (0.76, 1.36)	73.9	0.90 (0.62, 1.32)	79.4	1.02 (0.70, 1.49)
**Dental experience and attitude**								
** Children’s experience of abscess/infection**								
No	61.2	1	71.8	1	85.1	1	75.0	1
Yes	62.3	1.02 (0.73, 1.42)	74.8	1.04 (0.77, 1.42)	71.4	0.84 (0.56, 1.26)	83.3	1.11 (0.74, 1.66)
** The youngest child’s experience of dental treatment**								
Yes	50.0	1	65.7	1	69.5	1	65.2	1
No	66.3	1.33 (0.91, 1.93)	76.6	1.17 (0.83, 1.63)	82.3	1.18 (0.77, 1.81)	87.3	1.34 (0.87, 2.06)
** Children’s primary teeth**								
Should be filled	59.1	1	69.0	1	86.8	1	83.8	1
Are not worth treating/ should be removed	67.6	1.14 (0.82, 1.61)	83.8	1.21 (0.89, 1.65)	75.3	0.87 (0.57, 1.32)	76.8	0.92 (0.60, 1.41)

*PR* prevalence ratio, *CI* 95% confidence interval

## Discussion

To the best of our knowledge, this is the first study to report the parental acceptance of using SDF in Iran and Tajikistan. In summary, using SDF was widely acceptable by parents for all primary teeth in Iran (61.6%) and Tajikistan (77.9%). For permanent teeth, however, SDF was generally acceptable only for posterior teeth (Iran: 73.5%, Tajikistan: 78.7%), and thus was not supported for anterior teeth. Also, demographic characteristics and dental factors did not predict parental acceptance of using SDF. This may indicate that parents, regardless of their background and their children’s previous dental experience, made their decision.

We used a video to compare SDF and conventional methods of managing dental caries. This permitted parents to make an informed decision about the treatment plan rather than giving feedback on only the aesthetic acceptability of SDF. As a result, this research differs from similar studies which specifically highlighted the discolouration of SDF and asked parents to report its aesthetic acceptability [18, 24]. Our findings, therefore, reflect the choice of treatment taking into account the strengths and weaknesses of alternative options. We believe this is one of the strengths of our study as the acceptability of a health care intervention is a multi-faceted construct [25]. It is likely that the parents who may not be entirely pleased with the aesthetic appearance of SDF, consciously choose this treatment because of its strengths (e.g. its less invasive properties). There are review studies in which parental acceptance of SDF has been reported following the application of SDF on the children’s primary teeth [17, 26]. These studies also seem to report high acceptability of SDF after its application. In another study, 73% of American parents whose child received SDF reported they were not bothered by the SDF-related changes at all [27].

Further, the choice of treatment is an outcome of shared decision making between health care professionals and patients. It is therefore not surprising that the choice of SDF could be indirectly influenced by the perception of dental professionals. Three qualitative analyses of dental professionals’ perception toward SDF in Japan, UK and Switzerland reveal significant concerns about the aesthetic appearance of SDF treated teeth [28–30]. A scoping review of the literature suggested that dental professionals may be even more concerned than parents about the aesthetic acceptance of SDF [26]. The negative perception by dentists may inevitably lead to not offering SDF to patients.

At least one other study used a video successfully to educate parents on the characteristic of SDF prior to reporting its acceptability [31]. While the idea of ‘shared decision making’ has yet to be embraced in dentistry [32], the video we produced may facilitate the production of a decision aid for treating child patients in both countries.

In the 1970s when Japan was facing a shortage of dental workforce and high rate of child dental caries they incorporated the use of SDF within government dental care [28]. The renewed interest in application of SDF, as an inexpensive solution, can particularly benefit low-resource geographies and communities where there is a lack of dental workforce for managing childhood dental caries. The high rate of parental acceptance in this study may be regarded as a significant step toward promotion and adoption of SDF for community-based programs in Iran and Tajikistan. The less technique sensitive approaches such as SDF also pave the way for training non-dental health care professionals for getting involved in delivering dental care to children in such low-resource areas. The idea of task-shifting in dental care which means shifting the responsibility to those with shorter training period has been endorsed in the 2023 WHO global action plan. This document explicitly recommends developing ‘innovative workforce models’ and identifies the need to ‘revise and expand competency-based education to respond to population oral health needs’ [33]. Publication of a clinical guideline for non-invasive management of dental caries for non-dentists is an example of the opportunities SDF may offer [34]. Another example is the US study in which registered nurses were trained to deliver SDF treatment [35]. We reported the parental acceptance of SDF in two LMICs where access to dental care is limited and therefore SDF could offer an effective public health solution.

Our study had some limitations. Most notably, we used convenient sampling; therefore, no attempt was made to account for the socioeconomic and demographic characteristics of the participants. As a results, caution should be practiced when interpreting the finding of this study. We contemplated the idea of collecting clinical data on dental caries and adopting more robust sampling methods, however, this was not possible given the lack of resources and difficulty of working in these countries. While we did not collect clinical oral health data in this study, we recognise that the previous experience of dental diseases may affect parental acceptance of the SDF [36]. For practical reasons, we adopted different approaches for collecting data in Iran and Tajikistan. In Iran, where community health workers interviewed participants, the missing data were limited. In Tajikistan, however, some questions did not have valid responses due to the self-administered nature of data collection. For this reason, caution was practised for comparing the data from Iran with those from Tajikistan. Another limitation of the study is related to its exclusively quantitative design. For example, it is likely that, in addition to weaknesses and strengths of SDF which we included in this study, other factors had influenced the parental decisions. Qualitative studies are best placed to explore underlying reasons for the choice of treatment. There are at least three such studies in which parental views on SDF were investigated in the UK, Hong Kong and Canada [9, 37, 38]. These studies pointed to a range of reasons for declining SDF such as the possibility of toxicity [37] as well as of bullying at school due to black discoloration [9]. Furthermore, qualitative studies should explore similar and cultural reasons that may influence parental decision making.

## Conclusions

Establishing parental acceptance of SDF treatment is a fundamental step to its wider application among children. The present study showed that SDF was widely accepted by parents in both Iran and Tajikistan for all primary teeth and only posterior permanent teeth. Moreover, demographic factors and dental experience and attitude did not determine parental acceptance of using SDF.

## Data Availability

The datasets generated and/or analysed during the current study are not publicly available due to data sharing agreement but are available from the corresponding author on reasonable request.

## Abbreviations

SDF: Silver Diamine Fluoride
LMICs: Low-income and middle-income countries
WHO: World health organization
PRs: Prevalence ratios
CI: Confidence intervals
